# Identification and characteristics of patients with palliative care needs in Brazilian primary care

**DOI:** 10.1186/s12904-016-0125-4

**Published:** 2016-06-01

**Authors:** Fernando C. I. Marcucci, Marcos A. S. Cabrera, Anamaria Baquero Perilla, Marilia Maroneze Brun, Eder Marcos L. de Barros, Vanessa M. Martins, John P. Rosenberg, Patsy Yates

**Affiliations:** Postgraduate Program in Public Health, Universidade Estadual de Londrina, Londrina, Brazil; Department of Internal Medicine, Universidade Estadual de Londrina, Londrina, Brazil; School of Medicine, Universidade Estadual de Londrina, Londrina, Brazil; Institute of Health and Biomedical Innovation, Queensland University of Technology, Kelvin Grove, Queensland Australia; School of Nursing and Institute of Health and Biomedical Innovation, Queensland University of Technology, Kelvin Grove, Queensland Australia; Av. Robert Koch, 60. Vila Operaria, 86038-350 Londrina, Parana State Brazil

**Keywords:** Palliative care, Primary health care, Family health, Government Programs

## Abstract

**Background:**

The Brazilian healthcare system offers universal coverage but lacks information about how patients with PC needs are serviced by its primary care program, *Estratégia Saúde da Família* (ESF).

**Methods:**

Cross-sectional study in community settings. Patients in ESF program were screened using a Palliative Care Screening Tool (PCST). Included patients were assessed with Karnofsky Performance Scale (KPS), Edmonton Symptom Assessment System (ESAS) and Palliative Care Outcome Scale (POS).

**Results:**

Patients with PC needs are accessing the ESF program regardless of there being no specific PC support provided. From 238 patients identified, 73 (43 women, 30 men) were identified as having a need for PC, and the mean age was 77.18 (95 % Confidence Interval = ±2,78) years, with non-malignant neurologic conditions, such as dementia and cerebrovascular diseases, being the most common (53 % of all patients). Chronic conditions (2 or more years) were found in 70 % of these patients, with 71 % scoring 50 or less points in the KPS. Overall symptom intensity was low, with the exception of some cases with moderate and high score, and POS average score was 14.16 points (minimum = 4; maximum = 28). Most patients received medication and professional support through the primary care units, but limitations of services were identified, including lack of home visits and limited multi-professional approaches.

**Conclusion:**

Patients with PC needs were identified in ESF program. Basic health care support is provided but there is a lack of attention to some specific needs. PC policies and professional training should be implemented to improve this area.

**Electronic supplementary material:**

The online version of this article (doi:10.1186/s12904-016-0125-4) contains supplementary material, which is available to authorized users.

## Background

Chronic non-communicable diseases (NCD) are the main cause of death in the world and the number of people affected by NCDs is increasing [1, 2]. These conditions require progressive and continuous care, with timely access to palliative care (PC) recommended to ensure a better quality of life and support for families at end of life and in bereavement. As the disease progresses, patients require a range of health services, many of which are required while the patient remains living in the community [3].

For optimal care, the World Health Organization (WHO) has advocated for the role of PC early in the course of a disease, rather than solely in the end-stages. The WHO also recommends integration of services across all levels of health care, with an emphasis on primary care [3]. Currently, however, there is insufficient access to PC worldwide, especially in middle and low-income countries. Brazil, for instance, provides few PC services, located in the largest cities, but it covers a very limited amount of its population of over 200 million people. Moreover, access to this approach in the Brazilian public health system is concentrated to specialized cancer centers and local initiatives [4, 5].

Brazil offers universal health coverage in all levels of care by a decentralized network of health services, which includes community health units, ambulatory settings, and hospital care, free at the point of delivery to the entire population. However, limited resources can affect the quality of health care services and, although NCDs have a high impact on the Brazilian population and account for the majority of deaths, there are few specific policies for PC support in public health care system [4, 5]. As consequence, Brazil has a low rate of deaths at home or in nursing homes, and high occurrences in hospital settings, when compared with other countries [6].

The limited access to PC services in Brazil can reflect in a poor quality of dying, associated with intensive approaches and lack of multidimensional support. According to the Quality of Death Index published by The Economist Intelligence Unit [7], that ranked the availability, affordability and quality of end-of-life care in 80 countries, Brazil reaches the 42th position, and ranked below some coutries with similar or lower gross domestic product per capita, like Costa Rica, Jordan, South Africa and Cuba (middle income), and Mongolia and Uganda (low income).

The main Brazilian primary care policy is a community program called the Family Health Strategy (*Estratégia Saúde da Família/ESF*), which promotes preventive and health promotion interventions, chronic disease management and community care. This program has been associated with a decrease in hospitalization rates of chronic diseases [8]. However, there is a lack of information about primary health care services and professional support provided to patients with life-limiting conditions [9, 10].

To achieve a deeper understanding of the need for PC in Brazil, particularly in primary health care settings, the main objectives of this study were to:

- identify how many patients in the Brazilian ESF program have needs for PC;
- describe the health conditions and sociodemographic status of patients in the ESF program with PC needs; and,
- describe the professional and social support received by patients in the ESF program with PC needs.

## Method

A cross-sectional study was designed considering the organizational context of the Brazilian primary care services. The research was placed in the city of Londrina (State of Paraná), located in the South Region of Brazil. This is a mid-size city with high economic and social development for Brazilian standards, and regional reference for health care assistance. Initially, three community primary care units were selected for data collection. Each unit had two ESF teams and none of them offered any specific PC services, although the health care support included general practitioners (GP), nurses, nurses’ assistants and community health agents. These health agents are undergraduate trained workers responsible for registering, contacting, and developing health promotion activities and delivering basic orientation for a limited number of patients and families in a defined territory. Multi-professional supports teams (which includes nutritionist, physiotherapist, psychologist, social worker or other healthcare providers) were also available, of whom most are engaged in preventive and health promotion activities.

The ESF teams were asked to report all patients with potential need for PC, using instructions provided by the researchers with a pre-defined list with the most common conditions in PC, like advanced stage of heart and pulmonary diseases (with dyspnea on minimal exertion or at rest, long term oxygen therapy, continuous fatigue or other comorbidity); metastatic cancer or cancer with additional comorbidity; advanced human immunodeficiency virus disease; advanced liver or renal insufficiency disease; post-stroke with important functional impairments; dementia or frailty conditions [11, 12]. These professionals were asked to indicate all dubious case to be screened by the researchers.

All patients indicated by ESF teams were contacted by phone to schedule an appointment or were visited directly at their home address (if phone number was not available) to introduce the research purpose. If patients agreed to further contact, they were screened by the Palliative Care Screening Tool (PCST) from the Center to Advance Palliative Care (CAPC). Those who reached the score of 4 or more points should be considered to PC support. A score above 4 points indicates that the patient has additional aspects associated with end-of-life needs and reinforces the need for palliative assistance [13].

There is no “gold standard” tool to define a PC population, and sample selection for research and care provision can vary widely [14]. The PCST was chosen in this study due its objective approach to screening; ease and fast use; inclusion of non-malignant conditions; and because it is not limited only to functional status assessment. This tool seems useful for screening and with sensitivity to include patients with good functional status but with life-limiting related conditions. It does not replace professional evaluation and should not be used alone to classify PC patients for health care delivery [13, 15].

Selection criteria for inclusion were: adults (18 years or older), achieving four or more points on the PCST, being registered in the ESF program, and being willing to complete the Research Consent Form and assessment protocol. The patients included in the study were evaluated using a structured questionnaire with pre-defined questions about the patient’s clinical condition, social and demographic characteristics, caregiver relationship, services and professional supports received from the primary care unit in the last month. Functional status was evaluated by the Karnofsky Performance Scale (KPS) [16]. PC needs were raised by the Edmonton Symptom Assessment System (ESAS) and the Palliative Care Outcome Scale (POS/Portuguese version) [16, 17].

Identified patients whose contact details were outdated, who did not answer phone calls, or who were not found at home in two attempts were not included in further follow up. The data were collected from October 2014 to March 2015, and statistical analyses were performed with IBM SPSS Statistics 20 (IBM Corporation) and Microsoft Excel (Microsoft Corporation) softwares. Mean, 95 % confidence interval (CI) and percentage were used for descriptive statistical analyses. Analytic statistical procedures were performed to compare groups (independent *t*-test or Mann-Whitney *U* test), preceded by Kolmogorov-Smirnov test for normality distribution, and the significance level of 5 % (*p* ≤ 0.05) was considered for all statistical tests.

## Compliance with ethical standards

This research was conducted in the city of Londrina (Paraná State, Brazil) and was approved by the local department of health and by a research ethics committee registered by the Brazilian National Research Ethics Commission (Protocol: 17573113.8.0000.5231). The research protocol included the Research Consent Form and patients with cognitive impairment or unable to consent were represented by the legal carer.

## Results

**Fig. 1:**
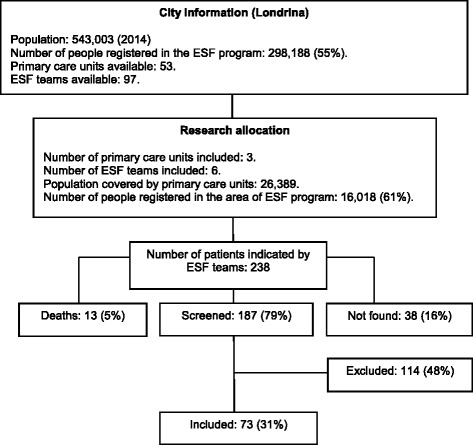
Locality information and sample selection flow chart for patients with need for PC in Family Health Strategy (ESF) program

Patients with identified needs for PC were found in all primary care ESF teams, that reported a total of 238 patients with potential PC needs, using instructions in the pre-defined list of conditions. Of these 238 reported patients, 13 died before the interview and a further 38 could not be located. Of the remaining 187 patients who were screened, 114 were excluded because they failed to meet inclusion criteria (3 or less points on the PCST, under 18 years old or diverse clinical conditions from the pre-defined list). Seventy-three patients met the inclusion criteria and there was no refusal to participate in the study. Patients’ selection and locality details are shown in Fig. 1.

**Fig. 2:**
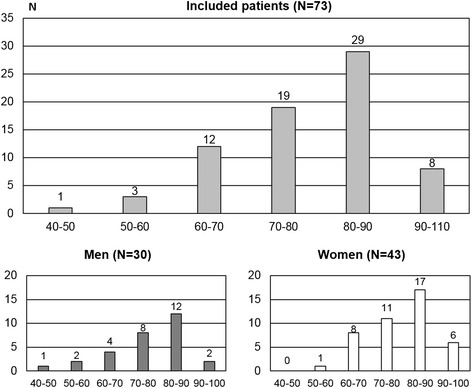
Age histogram of patients with need for PC in Family Health Strategy (ESF) program (general and gender groups)

Findings revealed that the frequency of patients with need for PC was 4.56/1,000 people registered in ESF program, and 5.37/1,000 if patients who died before the contact were included (considering the high probability of those to be considered as palliative care patients). Most patients included were elderly, having a mean age of 77.18 (95 % CI = ±2,78) years, (75.13 [95 % CI = ±5.21] for men and 78.6 [95 % CI = ±3.00] for women). There was no statistically significant difference for age between genders (*p* = 0.26). Age distributions for patients with PC needs are presented in Fig. 2.

Non-malignant diseases were the most common conditions amongst patients with PC needs, especially neurological conditions such as dementia related and cerebrovascular diseases (CVD), together comprising 53 % of this group. Chronic conditions were particularly common, as almost 70 % of the patients with PC needs had known the diagnosis of the main disease for two years or more years. Most patients indicated that they had their last hospital admission 6 months or more prior to data collection. Motor and cognitive impairments were identified as frequently affecting performance of daily activities, with 27 % of participant patients being totally dependent and 23 % totally incapable of providing any self-report information (Table 1).

**Table 1:** Clinical and social-demographic characteristics of patients with need for PC in Family Health Strategy (ESF) program

Main disease	Number	Percent
Dementia related diseases	20	27
Cerebrovascular disease (CVD)	19	26
Musculoskeletal disease	9	12
Others neurologic diseases	8	11
Congestive heart failure (CHF)	7	10
Cancer	6	8
Chronic renal failure (CRF)	2	3
Hepatopathy	1	1
Chronic obstructive pulmonary disease (COPD)	1	1
Secondary diseases (co-morbidity)		
None	24	33
One	44	60
More than one	5	7
Time from the last hospital admission		
Less than 1 month	3	4
From 1 to 3 months	8	11
From 3 to 6 months	9	12
More than 6 months (or none recently)	50	68
Do not know	3	4
Time since initial diagnosis		
Less than 6 months	5	7
From 6 to 12 months	4	5
From 1 to 2 years	11	15
More than 2 years	51	70
Do not know	2	3
Performance Status (ECOG performance status in PCST)		
0 – 1: Fully active or restricted in strenuous activity. Ambulatory and able to carry out work of a light or sedentary nature	3	4
2: Ambulatory and capable of all self-care but unable to carry out any work activities; up and about more than 50 % of waking hours.	24	33
3: Capable of only limited self-care; confined to bed or chair more than 50 % of waking hours	26	36
4: Completely disabled; cannot carry on any self-care; totally confined to bed or chair	20	27
Ability to provide self-information		
Totally capable	30	41
Partial capability with need for help of caregiver	20	28
Totally incapable	23	31
TOTAL	73	100

**Fig. 3:**
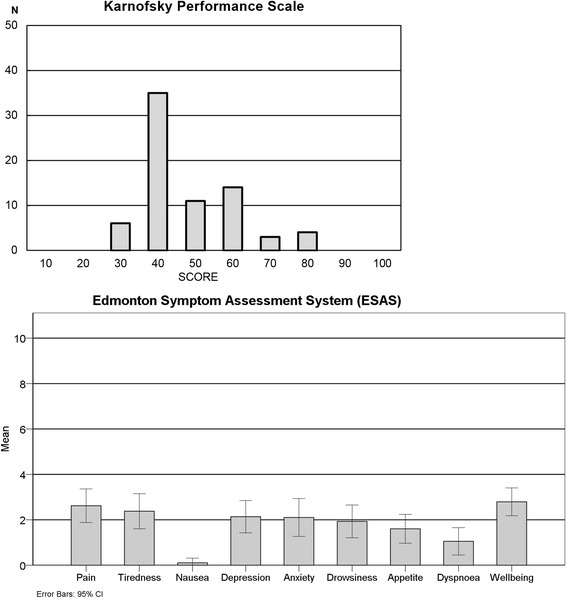
KPS and ESAS scores of patients with need for PC in Family Health Strategy (ESF) program

Low functional status is frequently used as being indicative of a need for PC [11]. Considering the different kind of diseases included, 71 % of patients with PC needs scored 50 or less using the KPS. Despite this, the use of PCST identified some patients with moderate to high functionality scores (over 50 points) but with other conditions associated with end-of-life conditions like having a progressive or incurable disease, multiple co-morbidities or several hospital admissions due the same problem. Overall, average symptom intensity was low although some patients reported moderate to high scores, assessed by ESAS. It should be noted that it was not possible to evaluate symptom intensity in some patients with cognitive impairment, particularly well-being, depression and anxiety (Fig. 3 and Additional file 1).

**Fig. 4:**
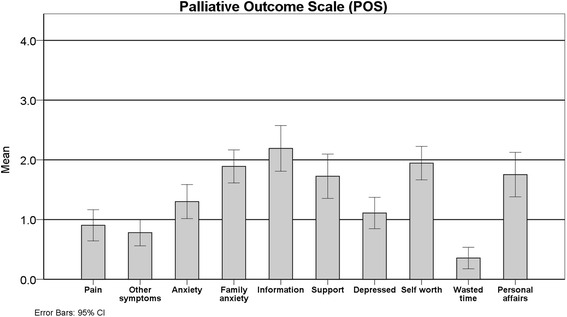
POS individual question scores of patients with need for PC in Family Health Strategy (ESF) program

Patients included were also assessed using the Palliative Care Outcome Scale (POS/Portuguese version). The average score was low to moderate, with a mean of 14.16 (95%CI = +1.47) points (Minimum = 4; Maximum = 28). Forty patients answered the self-report version (15 required help of the carer) and 33 were evaluated using the staff version, and there were no difference between these groups (*p* = 0.32). The most affected domain reflected the lack of information given to the patient and their family (Question 5), and symptoms were identified as mostly under control (Question 1, 2 and 9) (Fig. 4 and Additional file 1)

.

It was observed that most patients received medication (79 %) and professional orientation or consultation (59 %) from the primary care team, in the month prior from the interview. The professional support was provided mostly by nurses and nurses’ assistants (57 %), GPs (55 %) and community health agents (37 %) from the ESF teams included in the study, and it could be delivered at the patient’s home or in the primary care unit.

Despite having professional health coverage, there were some gaps in health care delivery as some patients had not received any support from primary care unit, or had less than one or no home visit from the ESF team, in the last month prior to data collection. Some limitations perceived by patients and families in primary care services were the long wait for scheduled appointments (examinations or professional consultations) and lack of home visits. For 41 % of participating patients, no problem in primary care unit services was cited.

Access to multi-professional approaches offered by primary care settings was very limited for these patients in the month prior to the interview. Only 10 % of the participant patients received physiotherapy, and none received psychology consult. These responses did not include the provision of this kind of assistance in private or other health care settings, however 76 % of patients reported that they did not have private health care insurance (Table 2).

**Table 2:** Professional health care and social support received by patients with need for PC in Family Health Strategy (ESF) program

Main service received by primary care unit	Number	Percent
Pharmacological drugs aquisition	58	79
Professional orientation/consult	43	59
Health supplies acquisition	8	11
Technical procedures	3	4
None	8	11
Received professional support		
Nurse	42	57
General Practioner	40	55
Community health agent	27	37
Physiotherapist	7	10
Social work	2	3
Nutritionist	1	1
Psychologist	0	0
None	17	23
Do not know	1	1
Home visit frequency		
1 or more times per week	1	1
2 to 3 times per month	12	16
Once per month	35	48
Less than 1 or none per month	24	33
Do not know	1	1
Main problem perceived in the primary care unit		
Lack of home visiting	23	31
Waiting time for appointment scheduling	10	14
Lack of professionals	6	8
Lack of material or drugs	4	5
None	30	41
Private healthcare ownership		
No	56	77
Yes	17	23
Marital status		
Married	32.00	44
Widower/Widow	29.00	40
Single	9.00	12
Divorced	3.00	4
Finance support		
Social insurance	68	93
Family or relative support	5	7
Institutional (non-governmental) support	0	0
Have a carer		
Yes	63	86
No	10	14
Carer gender		
Women	50	79
Men	13	21
Carer relationship		
Son/Daughter	30	41
Spouse	18	25
Mother	4	5
Brother/Sister	4	5
Grandson/Grandaugther	3	4
Other	2	3
Hired caregiver	2	3
None	10	14
Place of living		
Home	56	89
Other person house	7	11

Most patients were cared by a family member. Eighty percent of these carers were women with an average age of 60.87 (95%CI = ±3.75) years old. Married patients (44 %) and widowers or widows (40 %) were the most common matrimonial condition. Social insurance was the main source of financial support for the patients included. Further details of professional, social and familial support are shown in Table 2.

## Discussion

This study highlights the presence of patients with need for PC in the population registered in the ESF program despite there being no specific public health policies to provide PC in Brazilian primary care system [4]. According to the Global Atlas of Palliative Care at the End of Life [5], the estimated rate for adults with need for PC in the region of the Americas (including North, Central and South America) is about 353 to 365 per 100,000 habitants in the general population [5]. Gómez-Batiste et al. [18], screened the population from primary care settings in Catalonia (Spain), and found a prevalence from 1.1 to 1.4 % (1100 to 1400/100,000). Our study, found a frequency of 456 to 537/100,000 patients with need for PC registered in the Brazilian primary care (ESF program). These higher rates of PC patients found in primary care settings than from general population prevalence can be explained by the increased likelihood that patients facing incurable NCD or with end-of-life conditions will look for primary care surveillance to meet their heatlh needs, but further studies are necessary to identify the populational demands for PC services.

In this study, the patients included have an average age of 77 years old while life expectancy in Brazil is currently 74 years of age [19]. The higher average age and number of female patients is consistent with other studies findings since life expectancy tends to be higher in women [18]. These data highlight the importance of understanding how PC can be integrated into existing aged care services.

Dementia and CVD were the most frequent conditions of patients identified as needing PC. As a consequence, most patients had low functional status. A previous study by Luccheti et al. [15] has found similar results in a Brazilian nursing care setting. The chronic nature of these neurological conditions can be responsible for the elevated number of cases found in this community setting.

Cancer cases, on other hand, were low in the ESF program partly explained by the specific treatment settings for these patients in Brazil. Brazilian health policies for cancer management ensure that most cancer services are provided in specialized centers which do include PC assistance [20]. When referred for specific treatments, especially in end-stage conditions, they may lose the primary care surveillance offered by the ESF program as there is limited integration or shared care among health care services.

Integration of PC in the health care system is considered an important aspect to quality end-of-life care. According to the Global Atlas of Palliative Care at the End of Life [5], countries with high standard in PC support is characterized by the development of social activism; wide provision by multiple service providers; broad awareness of PC on the part of health professionals and society in general; unrestricted access to opioids and strong pain-relieving drugs; specific policies in this area, in particular on public health; educational and academic structure associated with PC development; and the existence of a related national association.

Currently, professional assistance in Brazilian primary care programs is provided by GPs, nurses and nurse’s assistants. In addition, the ESF program also includes Community Health Agents (*Agente Comunitário de Saúde*). These workers are responsible for monitoring clinical conditions of patients and making connections or arrange consultations with health care professionals, when necessary [21]. All primary care units also have a multi-professional teams called NASF (*Núcleos de Apoio à Saúde da Família*/Family Health Support Teams) which may include a psychologist, social worker, physiotherapist, occupational therapist, and other health professionals. These teams are responsible for individual or collective consultation, development of health promotion activities, and act as an advisory board for the ESF team. Despite some local initiatives, there are few national policies that promote PC delivery in primary care [21, 22].

The average intensity of many physical symptoms was low and the current treatment appeared to provide adequate control, despite a few cases with moderate and high intensity symptoms. Most physical symptoms were able to be managed through pharmacological treatment, and medication obtained from primary care setting was referred to by 80 % of participant patients. Medication access is an important issue in symptom management, and previous studies in Brazil have found that pharmacological support is widely provided in public health coverage [23]. Nevertheless, this study did not investigate if all prescribed medication, including opioids, were available in the primary care programs.

However, regardless of the availability of health care assistance, most patients did not have home visits from any health care team member in the month prior to the interview. Assessment of non-physical symptoms like well-being, depression and anxiety was also limited due to cognitive impairment and the subsequent difficulties in conducting evaluation with the chosen tools. Moreover, no included patients received psychological therapy, and very few had access to multi-professional support. Considering the large number of patients with cognitive and motor impairments, allied health professionals such as physiotherapists, social workers, nutritionists, could potentially provide important services to these patients.

The implementation of community-based PC, particularly in home services, have been associated with better symptom control, improvement in patient satisfaction, lower rates of hospital admission and lower costs than usual care [24–26]. These advantages need be explored in low and middle-income countries as an alternative to in-hospital based model, particularly for end-of-life support. The ESF program has been associated with decreased hospitalization rates, but Brazil still has a high rate of hospital deaths [6, 27, 28]. The implementation of community-based PC approach could have a mutual benefit for public hospital costs and patient satisfaction.

In recent years, the Brazilian government has launched a home care program to keep patients with low and medium-complexity conditions at home, and also as complementary support to primary care services. Although PC is included as one of its objectives, it is still not widely available [29, 30]. Accordingly, there is need for PC training for primary care professionals in order to improve the quality of care already provided by them and to address adequate support to specific PC needs.

Carers have an important role in health care support but are often vulnerable to physical symptoms, emotional distress, depression and anxiety development [31]. In this study, most carers labors in an informal activity (not-professional carers), and were women with an average age of 60 years old, similar to findings in other studies. Each informal carer have a different background, needs, physical capacity and health literacy level, so professional support strategies must consider these factors to plan specific interventions to promote improvements in the care delivered by those. Educational and behavioral interventions for carers have been associated with positive outcomes in anxiety control, care delivery competence, preparedness to act and feelings of reward [31]. Future research could investigate contextual, social and cross-cultural differences in carers support.

## Conclusion

This study aimed to provide information about patients with PC needs in Brazilian primary care settings. Chronic and non-malignant conditions, including dementia and CVD diseases, were the most frequent conditions identified in this population in the community setting. The ESF program offers some health care assistance for these patients, but there are no specific initiatives or policies to include PC support at this level of care. Most patients receive pharmacological treatment and basic medical and nursing assistance but a lack of multi-professional support was observed.

Non-professional support from families is very common and important for patients with PC needs, especially in community settings. The implementation of PC services should consider interventions to support the carers in care delivery and health assessment. Considering the increasing trend in NCD prevalence and population ageing, improvements in PC access is an urgent issue in public health in low and middle-income countries, like Brazil, and further studies are required to explore this framework. Despite the available health care support and some limited initiatives in PC development, improvements in the access to PC services to patients with life-limiting conditions and their families are required in the Brazilian health care system. To reach these improvements, specific public health policies should stimulate the PC development and define its role in NCD care; professional training structure need to be expanded; and social awareness and community engagement with PC issues must be debated.

## Abbreviations

CAPC, Center to Advance Palliative Care; CI, confidence interval; CVD, cerebrovascular diseases; ESAS, Edmonton symptom assessment system; ESF, Estratégia Saúde da Família/Family Health Strategy; GP, general practitioners; KPS, Karnofsky performance scale; NASF, Núcleos de Apoio à Saúde da Família/Family Health Support Teams; NCD, non-communicable diseases; PC, palliative care; PCST, palliative care screening tool; POS, Palliative care outcome scale; WHO, World Health Organization

## Additional file

Additional file 1: Frequencies of individual responses for ESAS and POS. These tables provide frequencies of individual responses for ESAS and POS for all included patient. (DOCX 15 kb)
