# Supplementary material for: Identification and characteristics of patients with palliative care needs in Brazilian primary care
Source: BMC Palliat Care. 2016 Jun 1;15:51. doi: 10.1186/s12904-016-0125-4 (PMC4888621; doi:10.1186/s12904-016-0125-4)
Supplement: Additional file 1: — Frequencies of individual responses for ESAS and POS. These tables provide frequencies of individual responses for ESAS and POS for all included patient. (DOCX 15 kb) [file 12904_2016_125_MOESM1_ESM.docx]

**Additional files**

**Additional file 1:** Frequencies of individual responses in ESAS e POS.

| Responses frequencies in Edmonton Symptom Assessment System (ESAS) | | | | | | | | | |  |
| --- | --- | --- | --- | --- | --- | --- | --- | --- | --- | --- |
| Score | Pain | Tiredness | Nausea | Depression | Anxiety | Drowsiness | Lack of appetite | Shortness of breath | Well-being | |
| 0 | 40 | 41 | 70 | 32 | 45 | 41 | 47 | 57 | 18 | |
| 1 | 0 | 2 | 1 | 2 | 0 | 3 | 3 | 0 | 2 | |
| 2 | 4 | 3 | 0 | 4 | 1 | 1 | 1 | 1 | 6 | |
| 3 | 6 | 6 | 0 | 4 | 4 | 6 | 4 | 8 | 8 | |
| 4 | 4 | 3 | 0 | 5 | 1 | 6 | 3 | 1 | 6 | |
| 5 | 7 | 8 | 1 | 6 | 5 | 5 | 8 | 1 | 12 | |
| 6 | 4 | 2 | 1 | 2 | 3 | 3 | 1 | 1 | 2 | |
| 7 | 3 | 1 | 0 | 2 | 4 | 4 | 2 | 1 | 4 | |
| 8 | 4 | 3 | 0 | 2 | 2 | 3 | 2 | 0 | 0 | |
| 9 | 0 | 0 | 0 | 0 | 2 | 0 | 1 | 1 | 0 | |
| 10 | 1 | 4 | 0 | 1 | 1 | 1 | 0 | 2 | 0 | |
| N/A* | 0 | 0 | 0 | 13 | 5 | 0 | 1 | 0 | 15 | |
| Mean | 2.2 | 2.2 | 0.2 | 2.1 | 2.0 | 2.1 | 1.6 | 1.1 | 2.8 | |
| SD^#^ | 2.9 | 3.1 | 0.9 | 2.7 | 3.0 | 2.8 | 2.5 | 2.4 | 2.3 | |
| *N/A: Not possible to assess. ^#^Standard deviation. | | | | | | | | | |  |

| Responses frequencies in Palliative Outcome Scale (POS) | | | | | | | | | | | | | | | | | | | | |  |
| --- | --- | --- | --- | --- | --- | --- | --- | --- | --- | --- | --- | --- | --- | --- | --- | --- | --- | --- | --- | --- | --- |
| SCORE  (Description) | | POS QUESTION SETTINGS | | | | | | | | | | | | | | | | | | | |
|  | | Pain | | Other symptoms | | Anxiety | | Family anxiety | | Information | | Support | | Life worthwhile | | Self worth | | Wasted time | | Personal affairs |  |
| 0 (Not at all) | 39 | | 38 | | 25 | | 9 | | 18 | | 24 | | 28 | | 6 | | 60 | | 28 | |  |
| 1 (Slightly) | 12 | | 17 | | 18 | | 20 | | 11 | | 16 | | 20 | | 26 | | 0 | | 0 | |  |
| 2 (Moderately) | 12 | | 14 | | 17 | | 22 | | 9 | | 5 | | 17 | | 17 | | 13 | | 26 | |  |
| 3 (Severely) | 10 | | 4 | | 9 | | 14 | | 9 | | 12 | | 5 | | 14 | | 0 | | 0 | |  |
| 4 (Overwhelmingly) | 0 | | 0 | | 4 | | 8 | | 26 | | 16 | | 3 | | 10 | | 0 | | 19 | |  |
| Mean | | 0.9 | 0.8 | | 1.3 | | 1.9 | | 2.2 | | 1.7 | | 1.1 | | 1.9 | | 0.4 | | 1.8 | |  |
| SD | | 1.1 | 0.9 | | 1.2 | | 1.2 | | 1.6 | | 1.6 | | 1.1 | | 1.2 | | 0.8 | | 1.6 | |  |
